# MicroRNA-200b is downregulated in colon cancer budding cells

**DOI:** 10.1371/journal.pone.0178564

**Published:** 2017-05-26

**Authors:** Kirsten Nguyen Knudsen, Jan Lindebjerg, Boye Schnack Nielsen, Torben Frøstrup Hansen, Flemming Brandt Sørensen

**Affiliations:** 1 Danish Colorectal Cancer Center South, Vejle Hospital, Part of Lillebaelt Hospital, Kabbeltoft 25, Vejle, Denmark; 2 Department of Clinical Pathology, Vejle Hospital, Part of Lillebaelt Hospital, Kabbeltoft 25, Vejle, Denmark; 3 Institute of Regional Health Research, University of Southern Denmark, Winsløwparken 19, Odense C, Denmark; 4 Bioneer A/S, Hørsholm, Kogle Allé 2, Hørsholm, Denmark; 5 Department of Oncology, Vejle Hospital, Part of Lillebaelt Hospital, Kabbeltoft 25, Vejle, Denmark; University of South Alabama Mitchell Cancer Institute, UNITED STATES

## Abstract

**Background:**

The microRNA-200 (miR-200) family acts as a major suppressor of epithelial-mesenchymal transition (EMT). Impaired miR-200 expression may lead to EMT initiation and eventually cancer dissemination. The presence of tumor budding cells (TBC) is associated with metastasis and poor prognosis, and molecular similarities to EMT indicate that these cells may reflect ongoing EMT. The aim of this study was to investigate the expression of miR-200b in budding cells of colon cancer and the relationship with the EMT-markers E-cadherin, β-catenin and laminin-5γ2.

**Material & methods:**

MiR-200b was investigated by *in situ* hybridization in 58 cases of stage II (n = 36) and III colon (n = 22) cancers with tumor budding. Expression of E-cadherin, β-catenin and laminin-5γ2 was examined by immunohistochemistry. A multiplex fluorescence assay combining miR-200b with cytokeratin and laminin-5γ2 was employed on a subset of 16 samples.

**Results:**

MiR-200b was downregulated in the TBC at the invasive front of 41 out of 58 (71%) cases. The decline was present in both mismatch satellite stable and instable adenocarcinomas. The majority of cases also showed loss of membranous E-cadherin and increased nuclear β-catenin in the TBC, while laminin-5γ2 expression was upregulated at the invasive front and in the tumor buds of approximately half the adenocarcinomas. However, the miR-200b decline was not statistically associated with the expression of any of the EMT-markers. The miR-200b decline was also documented by multiplex fluorescence. Fourteen out of fifteen cases showed a decrease in miR-200b expression in the majority of the TBC, but no obvious relationship between miR-200b and laminin-5γ2 expression was observed. Conclusion: The findings support the assumption of a miR-200b related downregulation in colon cancer budding cells. Whether miR-200b expression may be of clinical significance awaits further studies.

## Introduction

Epithelial-mesenchymal transition (EMT) is considered a key event in carcinoma dissemination [1]. During this process, the neoplastic, non-motile, polarized epithelial cell transforms into a mesenchymal phenotype. The acquisition of migratory and invasive capabilities allows it to move into the bloodstream and spread to distant organs and establish metastasis [1, 2]. The molecular changes during EMT include loss of cell junctions, down-regulation of the cell adhesion molecule E-cadherin, and increased expression of mesenchymal markers such as N-cadherin and vimentin[2]. Once the circulating carcinoma cell extravasates, phenotypic reversion enables it to colonize and expand.

In the recent years, microRNAs (miRNAs) have been implicated as promoters or suppressors of EMT[3]. Especially, the miR-200 family (miR-200a, miR-200b, miR-200c, miR-141, and miR-429) appears to exert an inhibitory effect on EMT in a number of cancers by repressing transcription factors zinc finger E-box binding homeobox 1 (ZEB1) and 2 (ZEB2). This maintains E-cadherin levels and the epithelial phenotype[4, 5]. Correspondingly, studies show that low miR-200 expression levels are associated with tumor progression and poor survival[4], while a possible oncogenic role of increased miR-200 expression has been associated with mesenchymal-epithelial transition[6].

Owing to similarities between EMT and tumor budding, the latter may represent a static histologic image of this dynamic process [7, 8]. Tumor budding is defined as a single cancer cell or small clusters of up to five cancer cells present in the invasive front of carcinomas. In colorectal cancer (CRC), 20–40% of all cases exhibit this growth pattern, which is associated with adverse features such as lymph node metastasis, distant metastasis, and poor prognosis[9]. Immunohistochemical (IHC) analyses have demonstrated that tumor budding cells (TBC) exhibit molecular traits such as loss of E-cadherin and EpCAM with accumulation of nuclear β-catenin and increased laminin-5γ2, similar to the changes found during EMT [10, 11]. However, knowledge on the miR-200 family in TBC of CRC is still scant.

The purpose of this study was to describe and examine miR-200b expression in colon cancer tumor buds in relation to EMT markers E-cadherin, β-catenin, and laminin-5γ2.

## Material and methods

### Clinical specimens

Tissue from 58 formalin-fixed paraffin embedded stage II (n = 36) and stage III (n = 22) colon adenocarcinomas diagnosed in the period of 2000–2008 were utilized in the present study. The material was obtained from the diagnostic pathology archive of Department of Clinical Pathology, Vejle Hospital.

The inclusion criteria were conventional adenocarcinomas pT3 with TBC. The histological slide showing the deepest invasion of the bowel wall was chosen. The presence of budding was initially ensured by assessment according to Danish National Guidelines, where tumor budding is defined as the presence of at least 10 buds with no more than four cells in one 20x objective. The assessment was carried out on cytokeratin stained (AE1/AE3) slides. Specimens with a cancer free surgical margin ≤ 1 mm and specimens from patients who had received acute surgery or had died within one month of surgery were not included.

Next, two senior pathologists allocated the 58 cases in low or high budding groups based on the 10 HPF method. According to this method, the number of buds is counted in ten fields of view at a 40x objective [12, 13]. Cases with an average of ≤ 10 buds were designated as low budding, >10 as high budding [13]. In a few cases of disagreement, a final grading was carried out by consensus. Perineural and vascular invasion were re-assessed in all tumor sections, while data on tumor differentiation and localization were obtained from the pathology reports. Mismatch repair protein expression analysis was conducted on the tumors if this had not been completed in the diagnostic process (Table 1). Information on subsequent development of distant metastasis was retrieved via medical charts.

**Table 1:** Clinico-pathological data for the study group.

Variable		Colon adenocarcinomas (n = 58) No. (%)
**Gender**		
	Female	39 (67)
	Male	19 (33)
**Age (years)**		
	Mean	73
	Range	49–98
**Localization**		
	Right colon	33 (57)
	Left colon	25 (43)
**Stage**		
	Stage II	36 (62)
	Stage III	22 (38)
**Mismatch repair protein**		
	Proficient	48 (83)
	Deficient	10 (17)
**Malignancy grade**		
	Low	4 (7)
	Moderately	42 (72)
	High	12 (21)
**Vascular invasion**		
	No	50 (86)
	Yes	8 (14)
**Perineural invasion**		
	No	48 (83)
	Yes	10 (17)
**Tumor budding**		
	Low	38 (35)
	High	20 (64)
**Recurrence, distant metastasis**		
	No	43 (74)
	Yes	15 (26)

The Regional Committees on Health Research Ethics for Southern Denmark approved the conduction of the study (ID# S-20120075) and granted a waiver of informed consent. The study was registered at the Danish Data Protection Agency, and The Danish Registry of Human Tissue Utilization was consulted before any tissue samples were investigated.

### Immunohistochemistry

IHC was performed on 4 μm thick, adjacent sections involving AE1/AE3, E-cadherin, β-catenin, laminin-5γ2, and mismatch repair proteins MLH1, PMS2, MSH2, and MSH6. Heat-induced epitope-retrieval was achieved with Target Retrieval Solution, pH9 (Dako, Glostrup, Denmark, code K8004), the primary antibodies diluted with Envision FLEX antibody Diluent (Dako, code S2022) and incubated at room temperature as stated in S1 Table. The staining procedure was executed on a Dako Autostainer Plus (Dako) using EnVision FLEX+, Mouse, High pH, (Link) (Dako, code K8002).

### Evaluation of immunostatus

E-cadherin and β-catenin expression in TBC were evaluated in 100 buds from hot spots and it was noted whether the dominant staining pattern changed from the luminal half of the cancer toward the invasive front. Regarding E-cadherin, tumors were considered negative if at least 50% of the tumor buds presented with weak *membranous* staining or partly-completely loss of membranous stain +/- increased *cytoplasmic* reaction compared to the tumor center.

Tumors with >5% *nuclear* β-catenin stained budding cells were classified as positive.

Laminin-5γ2 was scored according to Shinto *et al*. [14]. In brief, tumors with the presence of >20% *cytoplasmic* immunopositive carcinoma cells at the invasive front, which included the TBC and the outermost cell rows at the invasive front, were categorized as laminin-5γ2 positive. β-catenin and laminin-5γ2 were evaluated based on percentage of positivity disregarding staining intensity as this approach may give a more accurate and reproducible assessment [15].

### Chromogenic *in situ* hybridization

*In situ* hybridization (ISH) for miR-200b was essentially performed as described previously [16]. Prior to the analysis, assay optimization was accomplished to determine optimal probe concentrations and hybridization temperatures. The ISH analysis was performed using a Tecan Evo automated hybridization instrument (Tecan, Switzerland). The following steps were performed on 6 μm thick sections: pre-digestion with proteinase-K (15 μg/ml) at 37°C for 8 minutes, pre-hybridization at 55°C for 15 minutes, followed by hybridization with 40 nM double-carboxyfluorescein (FAM) labelled Locked Nucleic Acid miR-200b-3p (sequence TCATCATTACCAGGCAGTATTA) (Exiqon, Vedbæk, Denmark) for 90 minutes. After stringent washes with saline-sodium-citrate buffer, the miR-200b probe was detected with alkaline phosphatase-conjugated sheep anti-FAM Fab fragments (Roche, Basel, Switzerland). Next, incubation with 4-nitro-blue tetrazolium and 5-brom-4-chloro-3′-Indolyl-phosphate substrate (Roche) for 2 hours resulted in a dark-blue precipitate, and the slides were finally counterstained with nuclear fast red.

### Evaluation of miR-200b ISH signal

The evaluation of miR-200b expression was carried out assuming equal thickness of the investigated tissue sections. The slides were scored semi-quantitatively with a 20x objective according to relative intensities (0 = negative, 1 = weak, 2 = strong) and proportion of positivity (0 = <10%, 1 = 10%-50% and 2 = >50%) in the tumor center, at the invasive front and for the TBC. The total score was determined by adding the intensity and proportion scores, which was then divided into two categories: low miR-200b expression, score 0–2, and high miR-200b, score 3–4 (S1 Fig).

### Multiplex fluorescence staining

Paraffin sections were cut at 5μm, air-dried and deparaffinized using xylene and ethanol solutions. The combined ISH and IHC fluorescence staining procedure is described in detail elsewhere [17]. In brief, sections were treated with proteinase-K using 25μg/ml for 10 minutes at 37°C. Sections were hybridized with a double-FAM-labeled LNA probe for miR-200b-3p (5’TCATCATTACCAGGCAGTATTA’3; RNA Tm, 85^°^C; 32% LNA) at 40nM in Exiqon hybridization buffer (Exiqon) at 52°C for 2 hours. The probe was detected with peroxidase-conjugated anti-FAM (Roche) followed by incubation in TSA-Cy5 substrate (Perkin Elmer, Waltham, MA, USA) for 10 minutes at room temperature. Slides were washed in PBS and incubated for 10 minutes with 3% hydrogen peroxide. Hereafter, two sequential immunofluorescence staining procedures were performed. First, mouse-anti-cytokeratin, clones AE1/AE3, was incubated on sections (diluted 1:200, Dako) overnight at 4°C, and detected with horseradish peroxidase-conjugated anti-mouse (Jackson ImmunoResearch, West Grove, PA, USA) followed by incubation in TSA-FITC substrate (Perkin Elmer) for 7 minutes at room temperature. After brief washes in PBS, sections were treated with glycin/SDS-buffer to elute all antibodies from the sections [18]. Next, mouse-anti-laminin5 (γ2 chain), clone D4B5 (diluted 1:200, Merck Millipore, Billerica, MA, USA) was incubated on sections at room temperature, and detected with Cy3-conjugated anti-mouse (Jackson ImmunoResearch). Sections were mounted with DAPI-containing mounting medium, ProLong Gold (Thermo Fisher Scientific, Waltham, MA, USA). The subsequent days, sections were investigated and images acquired with a 20x objective using an epifluorescence microscope (AxioImager, Oberkochen, Zeiss, Germany) equipped with an HXP 120 V Illuminator and the following relevant filter sets: Set 49/Dapi G365-BP445/50, Set 36-HE/FITC BP470/40-BP525/50, Set 43-HE/Cy3 BP550/25-BP605/70, and Set 50/Cy5 BP640/30-BP690/50. Images were acquired from the tumor periphery region, where cytokeratin-positive TBC were evident. Areas with excess autofluorescence signal were avoided. Exposure time was that of the auto-threshold determined by the Zeiss software for each of the four fluorescence signals. Images were post-processed for balanced signal-to-noise.

### Evaluation of the multiplex fluorescence assay

MiR-200b was evaluated in green cytokeratin-positive TBC as well as in cohesive cancer tissue. The cells were designated as low or high expressing based on signal intensity. A similar approach was used for laminin-5γ2 (red signal) evaluation in the TBC. The cells were considered to express high laminin-5γ2 in the presence of a yellow signal as a result of green and red merge, low expressing if the signal was primarily green. In some slides a white signal was identified in some stromal leukocyte-like cells. This was considered to represent autofluorescence and not true miR-200b ISH signal after inspection of the respective chromogenic miR-200b stained sections, where no such ISH signal was found.

### Statistical analysis

STATA version 14.0 (StataCorp, College Station, TX, USA) was used for all statistical analyses. Fisher’s exact test examined the possible associations between miR-200b expression and clinico-pathologic characteristics and the expression of the EMT-markers. P-values ≤0.05 were considered statistically significant.

## Results

### EMT markers in tumor budding cells

Whole tissue slides, stained by IHC, were scored for E-cadherin (n = 56), β-catenin (n = 52) and laminin-5γ2 (n = 58); the missing cases were excluded due to absence of tumor budding in deeper sections of the tissue block.

**Fig 1:**
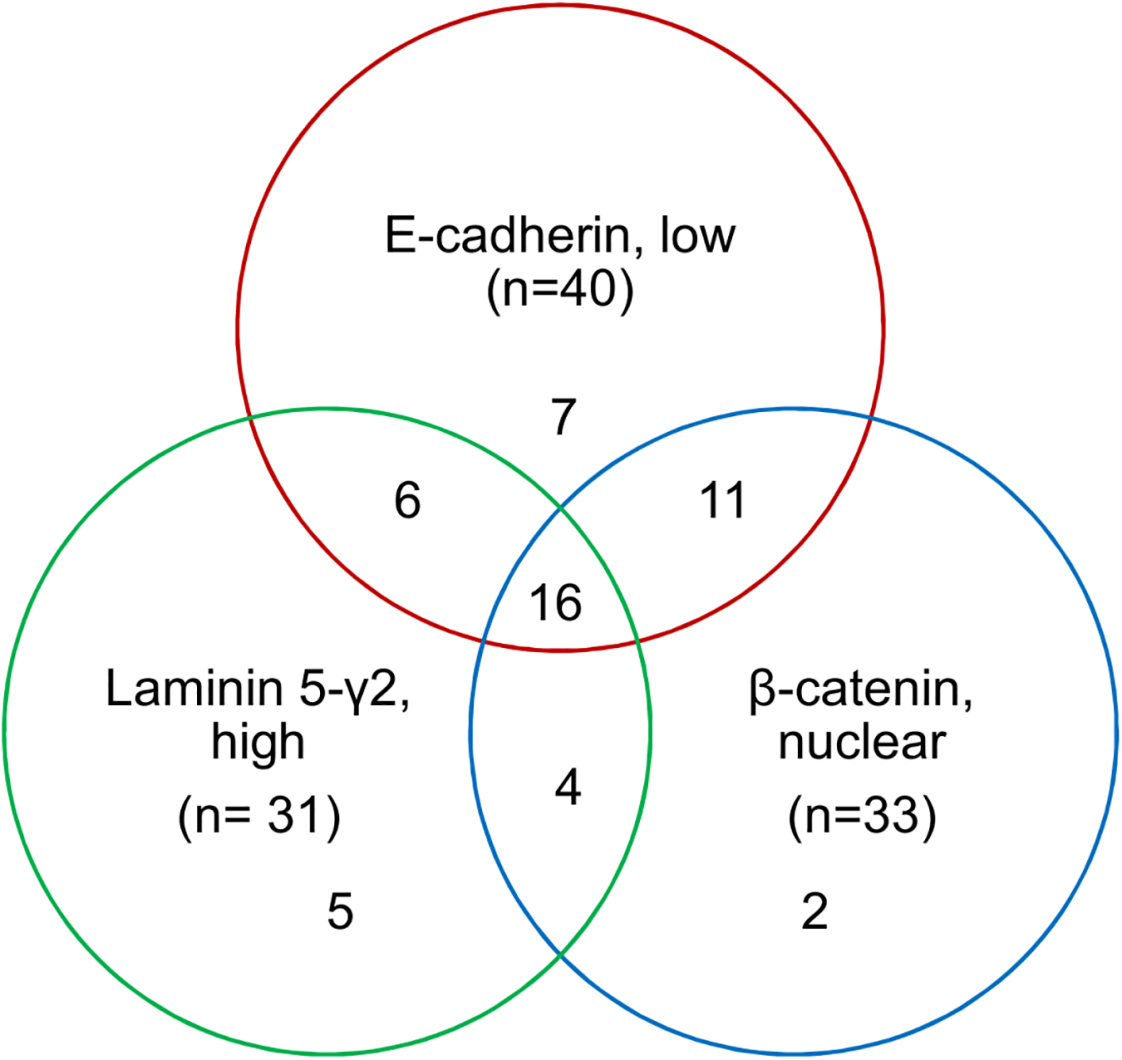
EMT-protein distribution in tumor center and at the invasive front. Venn diagram of colon cancers with tumor budding showing differentially expressed E-cadherin, nuclear β-catenin, and laminin-5γ2 in the invasive front compared to the tumor center. Out of 58 cases, a total of 51 cancers showed changes in at least one marker.

All tumors showed weak to moderate membranous E-cadherin staining in the tumor center. At the invasive front, 40 cases (71%) exhibited a pronounced loss of membranous E-cadherin staining and increased cytoplasmic staining in the TBC. β-catenin expression changed from a chiefly membranous staining with or without cytoplasmic reaction in the tumor center to nuclear accumulation in the budding cells in 33 cases (63%). Thirty one cases (53%) showed cytoplasmic laminin-5γ2 upregulation at the invasive front and in tumor buds (S2 Fig). Sixteen cases showed simultaneous changes of all three proteins, 21 cases displayed changes in two EMT markers, and 14 cases in one marker (Fig 1). Seven out of 58 (12%) tumors had no evidence of EMT change. It was noted that five of these were mismatch repair protein deficient cancers (71%), whereas this was the case for only five out of 51 (10%) cancers displaying EMT (*p* = 0.001). This could be attributed to the expression of β-catenin alone, in that nuclear β-catenin expression was absent in the MSI TBC, but present in the mismatch satellite stable (MSS) TBC (p<0.001). The levels of E-cadherin and laminin-5γ2 did not differ significantly between the two groups.

### MiR-200b expression in colon cancer

The miR-200b ISH signal in chromogen stained slides varied from case to case. Examples of miR-200b staining are shown in S1 Fig. When present, the signal was overall confined to epithelial cells. Based on a semi-quantitative estimation with a cut-off score ≤ 2, the clinical samples were divided into low-expressing (n = 29) and high-expressing (n = 29) subgroups, according to their central tumor miR-200b expression (Table 2). No statistical associations with the EMT markers, high budding, or other clinico-patological variables were found (*p*>0.15).

**Table 2:** MiR-200b expression in the tumor center, invasive front and the tumor budding cells.

	Tumor center	Invasive front	Tumor budding cells
N	58	57^a^	58
Low miR-200b	29	36	41
High miR-200b	29	21	17

^a^Data missing due to tissue tearing at the invasive front

Low miR-200b expression was found in the invasive front of 36 colon adenocarcinomas. Ten cases from the high expressing subgroup exhibited reduction of the miR-200 signal from the central tumor area to the invasive front, while three cases from the low expressing subgroup showed increased signal toward the invasive front. No obvious decline was seen in the remaining 19 cases.

**Fig 2:**
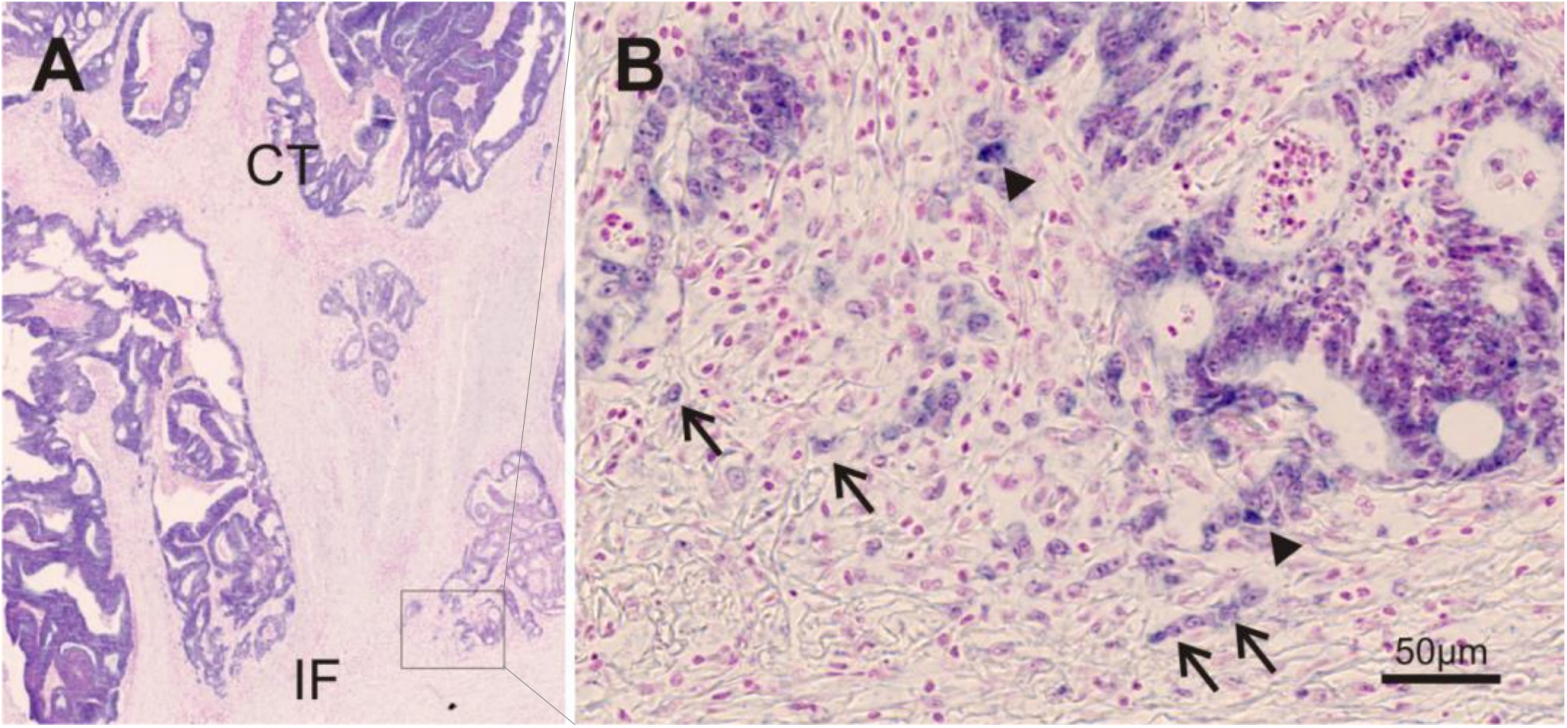
MiR-200b expression in the tumor center and invasive front of colon cancer. **(A)** High miR-200b expression is present in the central part of the tumor (CT), while **(B)** expression is decreased in several tumor budding cells (arrows) at the invasive front. High expression is present in only few buds (arrowheads). The image also illustrates that the identification of tumor budding cells in a CISH stain may be challenging. The cells are easily identified, when miR-200b expression is high, but much more difficult, when miR-200b is decreased. Single cells or small groups of cells with large nuclei and distinct nucleoli may suggest tumor buds, but unambiguous identification and quantification on a CISH stain is impossible.

Forty-one cases (71%) out the 58 cases displayed low miR-200b expression in the TBC (Fig 2). No statistically associations to the changes of E-cadherin, β-catenin or laminin-5γ2 (*p*>0.07) were found. However, when the miR-200b signal was not markedly present in the tumor buds, the cells were difficult to identify with certainty in the ISH-stained slides despite the nuclear red counterstain. This was not surprising, because TBC sometimes requires cytokeratin IHC for unambiguous identification. A subset of cases was therefore submitted to multiplex fluorescence analysis to substantiate the observations.

#### Decreased miR-200b is found in tumor budding cells by multiplex fluorescence

To achieve a close examination of the miR-200b signal in the tumor buds, a subset of 20 samples was chosen for a triple immunofluorescence analysis, combining pancytokeratin and laminin-5γ2 IHC with miR-200b ISH. The sample subset was based on: 1) high degree of budding; 2) cases with and without metastatic progression (ten of each); and 3) tumors with (n = 8) and without (n = 12) miR-200b positive TBC in the chromogenic ISH analysis. The selection included tumors from both the low and high miR-200b expressing groups. Four cases were excluded due to tissue detachment during the staining procedure. A fifth case did no longer display TBC.

**Fig 3:**
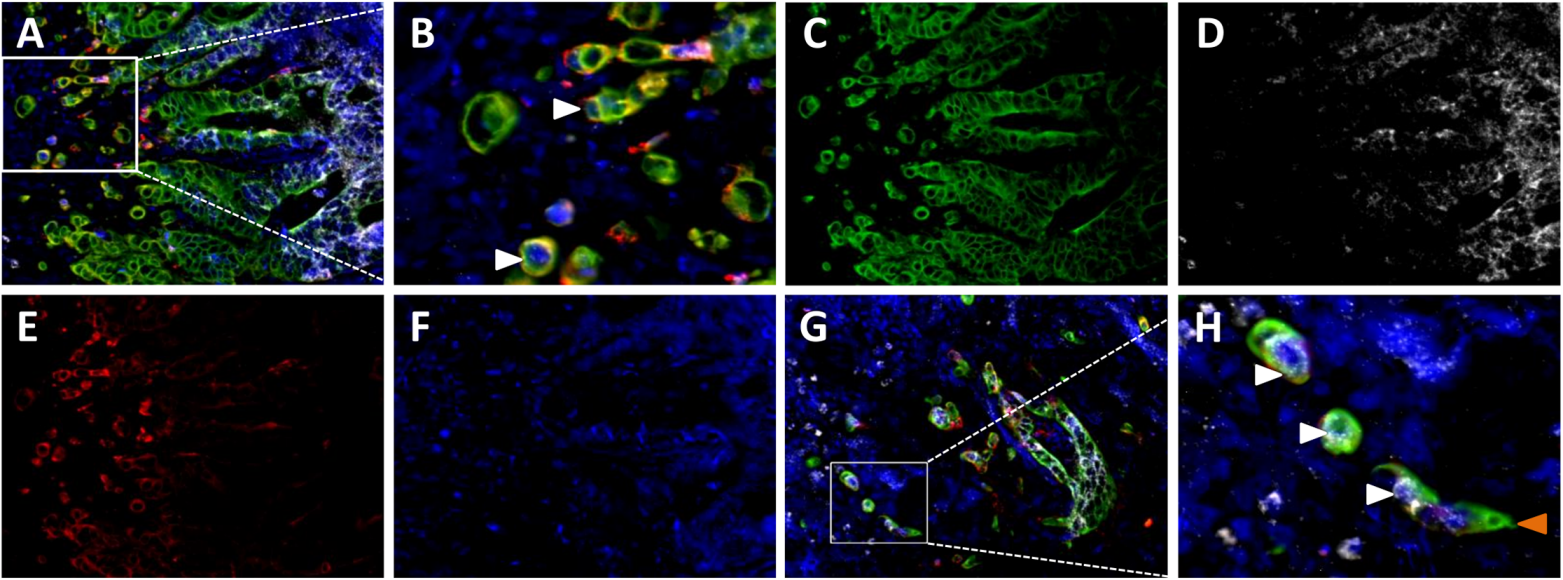
Triple stain for miR-200b, AE1/AE3, and laminin-5γ2 at the invasive front. **(A)** Merged image: cytokeratin AE1/AE3 (green), miR-200b (white), and DAPI (blue). **(B-F)** Enlarged area from A. **(B)** Tumor budding cells co-expressing cytokeratin AE1/AE3 and laminin-5γ2 (red) appear yellow as indicated by white arrowheads. **C)** Immunofluorescence for cytokeratin AE1/AE3 identifying cytokeratin-expressing carcinoma cells. (**D)** A strong miR-200b signal is present in the luminal part of the invasive front, but diminishes gradually towards the outmost periphery and the budding cells. **(E)** The inverse pattern is seen for laminin-5γ2. **(F)** DAPI. (**G+H)** Merged images from a different tumor displaying retained miR-200b expression in some budding cells (white arrowheads) and decreased levels in others (orange arrowhead). Scale bars 50 μm (A, C, D, E, F, G), and 25 μm (B, H).

As expected, a specific miR-200b signal was found in cytokeratin-positive cells. The miR-200b intensity was strong in the cohesive cancer tissue, but decreased at the edge of the invasive front, and especially in the tumor buds (Fig 3). MiR-200b was downregulated in all fifteen cases in the majority of the examined TBC (mean 80%, range 21–100%). The decrease was seen in both MSS and MSI adenocarcinomas. The percentage of budding cells with concomitant low miR-200b and high laminin-5γ2 expression (mean = 39%, range 0–94%), and cells with simultaneously low miR-200b and low laminin-5γ2 expressions (mean = 42%, range 6–100%) was similar, thus no obvious relation between miR-200b and laminin-5γ2 expression was observed (S2 Table). No difference between the staining patterns of miR-200b or laminin-5γ2-patterns in cases with and without subsequent recurrence was observed.

## Discussion

The miR-200 family plays an essential role in EMT, primarily through direct suppression of the ZEB1/2 transcription factors, affecting a number of genes associated to the epithelial and mesenchymal phenotype [5, 19]. In turn, ZEB also controls the entire miR-200 family by negative feedback. An imbalance in this double-negative feedback loop in epithelial neoplasms is assumed to activate EMT in the tumor periphery and possibly mesenchymal-epithelial transition in established metastases [20]. Dysregulated miR-200 and ZEB levels have been found in many different cancers [4, 20]. ZEB1 is upregulated at the invasive front of CRC, primarily in the stromal cells, whereas expression in the dedifferentiated adenocarcinoma cells is discrete [21–23]. As TBC are recognized as the histologic snapshot of EMT in progress, they provide an opportunity to study the miR-200-family in clinical samples.

The results showed decreased miR-200b signal in colon cancer budding cells in adenocarcinomas presenting with both high and low budding patterns. At present, the literature on microRNA-expression in TBC is limited. To our knowledge, an examination of a member of the miR-200 family in colon cancer budding cells has hitherto not been reported. In oral squamous cell carcinoma, Jensen *et al*. employed qPCR array on laser micro-dissected tumor buds and showed that the expression of all five miR-200 family members were decreased compared to central tumor; miR-200c and miR-141 were further validated with ISH [24]. In contrast, decreased levels of the miR-320 family, but not the miR-200 family, were observed in budding cells of tongue squamous cell carcinoma pointing toward tumor type specific patterns of miRNA expression[25].

Focusing on the invasive front, Paterson *et al*. found decreased miR-200b and miR-200c in colorectal adenocarcinomas with evidence of budding and degraded basement membrane[21]. However, whether the expressions of the miRNAs were changed in the TBC was not reported. Hur *et al*. observed decreased miR-200c ISH signal at the invasive front in late stage CRC [26], while this was not observed by another research group [27]. Paterson *et al*. found that all 18 tumors with tumor buds and degraded basement membrane displayed declining changes in miR-200 in the tumor periphery [21] This was only observed in 10 out of 58 CISH-stained tumors in this present study. The criteria for tumor budding was not specified in the former study, thus differences in tumor budding evaluation as well as different T-and M-stages might account for the discrepancy. However, it is more likely that the different evaluation approaches played a significant role. Image analysis, as employed by Paterson *et al*., may objectively be able to measure intensity changes that could not be identified with our subjective, semi-quantitative approach, which was based on a dichotomous classification. Thus, in our study only a change from high to low expression was recorded as a declining pattern. The advantage of our approach, however, was the assessment of the entire tumor periphery reducing the risk of sampling bias.

Since TBC are considered in a transitional stage of ongoing EMT, the inclusion of these cells, if present, in the investigation of the invasive front could lead to additional knowledge on the potential role of miR-200b loss in cancer progression. Not surprisingly, the TBC were far more difficult to distinguish in a CISH stain than in a H&E slide. However, even in H&E stained slides tumor buds may be difficult to identify from stromal cells with certainty[28]. In the present study, the multiplex analysis included cytokeratin to identify the TBC, providing unambiguous evidence of declining miR-200b expression at the invasive front, but in particular in the budding cells. It was also documented, that the miR-200b reduction was present in cases with both low and high miR-200b expression found by permanent chromogen ISH. Thus, the multiplex fluorescence staining demonstrated a highly advantageous method to characterize miRNAs in a specific cell type. The challenge could be finding an antibody compatible with the Proteinase K antigen retrieval approach, a prerequisite for the analysis.

In this study, a triple fluorescence assay combining pan-cytokeratin, miR-200b, and laminin-5γ2 was also used to evaluate the dynamic changes of miR-200b and laminin-5γ2 at the invasive tumor front with special attention on tumor buds. Although not a putative target of miR-200b, increased laminin-5γ2 expression at the tumor periphery of CRC correlates with invasion, metastasis and poor survival [14, 29, 30]. Furthermore, its gene, *LAMC2*, is activated by ZEB1 [31]. We therefore hypothesized that colon cancers from patients, who later developed distant dissemination, would exhibit an increased number of cells with concomitant miR-200b decrease and laminin-5γ2 increase compared to those with no progression, but no distinct pattern was observed. A possible explanation could be the small sample size or the heterogeneity of the studied cases. Although we sought to find cases with similar characteristics, including the same histologic variant and equal pT-stage, we could not adjust for other varying traits in this clinical material such as tumor stage, differentiation, localization, and mismatch repair protein status, where other molecular pathways may be dominant.

The majority of cancers showed signs of an ongoing EMT-process in terms of changed expression in at least one of the EMT markers E-cadherin, β-catenin, and laminin-5γ2, as described by others [10]. Simultaneous changes of all the markers were present in less than 30% of the cases. One reason could be due to the selected cut-off values for the markers utilized in the study. Another reason could be the combination of stage II and stage III colon cancers. As tumor budding is associated with lymph node metastases[32] and EMT is associated with tumor progression and metastasis[2], optimally, the study should have focused on the stages separately. However, since a sufficient number of cases matching the inclusion criteria could not be obtained, the groups were combined. The combination of a non-metastatic and a metastatic group could explain why some of the cases did not displayed changes in all markers. The majority of the cases did show changes in at least one pseudo-marker, indicating that EMT is likely initiated in TBC. Most of the cases that did not display these changes were MSI adenocarcinomas. The lack of increased nuclear accumulation of β-catenin in the MSI cases are similar to the findings in other studies[33, 34] and supports the assumption that EMT could be impaired in MSI CRCs [35]. It would be interesting to pursue this observation further and to extend such an investigation with examination of other EMT-related markers than those examined in this study. Interestingly, the miR-200b decrease was observed in the TBC of both MSS and MSI adenocarcinomas, implying that the miR-200b downregulation also occurs in colon adenocarcinomas with putatively impaired EMT. However, this observation was based on a small number of cases and should be confirmed in larger studies.

In conclusion, our descriptive study addressing a cell biologic process shows that miR-200b is expressed in central tumor areas of colon cancer and that expression decreases in the TBC at the invasive front. To our knowledge, this is the first study to provide visual evidence that miR-200b expression is reduced in TBC identified in clinical samples of colon cancers. Along with the molecular profile established with IHC over the years, our findings support the assumption of an EMT-like process in colon cancer budding cells. Whether miR-200b expression may be of clinical significance awaits further studies.

## Supporting information

S1 Table Detailed information on the primary antibodies used for IHC. (DOCX)

S2 Table The expression of miR-200b and laminin-5γ2 in the budding cells of the multiplex stained cases. miR-200 and laminin-5γ2 expressions were evaluated in the cytokeratin-positive tumor budding cells in the second column. miR-200b expression was considered low in the event of ≤50% positively stained cells, laminin-5γ2 as high if the proportion of positive cells exceeded 20%. The mean proportion of cells with combined ↑laminin-5γ2 and ↓ miR-200b expression was 39% (range 0–94%) and cases in the last column were allocated as low and high according to fractions below or above mean.
(DOCX)

S1 Fig Examples of miR-200b tumor stains. **(A)** Low miR-200b expression and **(B)** high miR-200b expression in the epithelial carcinoma cells of colon cancer.
(TIF)

S2 Fig Changes in EMT-markers in colon cancer tumor buddings cells. **(A)** Declining E-cadherin expression from central tumor (CT) towards the invasive front (IF) where **(B)** tumor buds show decreased membrane expression (arrows). **(C)** β-catenin expression changes from a chiefly membranous and cytoplasmic pattern to **(D)** nuclear localization in the tumor buds (arrows), while **(E+F)** laminin-5γ2 is upregulated at the invasive front and the tumor buds (arrows).
(TIF)

S1 Data Data from IHC and ISH analyses. (XLSX)
