# Supplementary material for: MicroRNA-200b is downregulated in colon cancer budding cells
Source: PLoS One. 2017 May 26;12(5):e0178564. doi: 10.1371/journal.pone.0178564 (PMC5446202; doi:10.1371/journal.pone.0178564)
Supplement: S1 Table — (DOCX) [file pone.0178564.s001.docx]

Table S1. Detailed information on primary antibodies used for IHC

| Antibody | Dilution | Incubation time, minutes | Provider | Product code |
| --- | --- | --- | --- | --- |
| AE1/AE3^[[1]](#endnote-1)^ | 1:250 | 30 | Dako | M3515 |
| β-catenin | 1:400 | 60 | Abcam | Ab32572 |
| E-cadherin | 1:100 | 30 | Dako | M3612 |
| Laminin-5 γ2 | 1:300 | 30 | Dako | M7262 |
| MLH1^[[2]](#endnote-2)^ | 1:100 | 30 | Novocastra | NCL-L-MLH1 |
| MSH2^[[3]](#endnote-3)^ | 1:100 | 30 | Novocastra | NCL-MSH2 |
| MSH6^[[4]](#endnote-4)^ | 1:200 | 30 | BD Biosciences | 610919 |
| PMS2^[[5]](#endnote-5)^ | 1:500 | 30 | BD Biosciences | 556415 |

1. Cytokeratin clone AE1/AE3 [↑](#endnote-ref-1)
2. MLH1: mutL homolog 1 [↑](#endnote-ref-2)
3. MSH2: mutS homolog 2 [↑](#endnote-ref-3)
4. MSH6: mutS homolog 6 [↑](#endnote-ref-4)
5. PMS2: PMS1 homolog 2 [↑](#endnote-ref-5)
