# Supplementary material for: MicroRNA-200b is downregulated in colon cancer budding cells
Source: PLoS One. 2017 May 26;12(5):e0178564. doi: 10.1371/journal.pone.0178564 (PMC5446202; doi:10.1371/journal.pone.0178564)
Supplement: S2 Table — miR-200 and laminin-5γ2 expressions were evaluated in the cytokeratin-positive tumor budding cells in the second column. miR-200b expression was considered low in the event of ≤50% positively stained cells, laminin-5γ2 as high if the proportion of positive cells exceeded 20%. The mean proportion of cells with combined ↑laminin-5γ2 and ↓ miR-200b expression was 39% (range 0–94%) and cases in the last column were allocated as low and high according to fractions below or above mean. (DOCX) [file pone.0178564.s002.docx]

| **Case** | **MMP status** | **Total TBC count** | **miR-200b low TBCs (%)**^a^ | **miR-200b low and**  **laminin-γ2 high TBCs (%)**^a^ | **miR-200b low and**  **laminin-γ2 low TBCs (%)**^a^ |
| --- | --- | --- | --- | --- | --- |
| 14 | MSS | 18 | 18 (100) | 15 (83) | 3 (17) |
| 19 | MSS | 17 | 17 (100) | 16 (94) | 1 (6) |
| 7 | MSS | 24 | 21 (88) | 1 (4) | 20 (83) |
| 8 | MSS | 49 | 43 (88) | 26 (53) | 17 (35) |
| 4 | MSS | 85 | 73 (86) | 36 (42) | 35 (41) |
| 16 | MSS | 51 | 44 (86) | 23 (45) | 21 (41) |
| 1 | MSS | 53 | 39 (74) | 22 (42) | 17 (32) |
| 9 | MSS | 41 | 30 (73) | 22 (54) | 8 (20) |
| 17 | MSS | 28 | 19 (68) | 4 (14) | 15 (54) |
| 3 | MSS | 61 | 35 (57) | 24 (39) | 11 (18) |
| 13 | MSS | 33 | 7 (21) | 4 (12) | 3 (9) |
| 12 | MSI | 8 | 8 (100) | 0 (0) | 8 (100) |
| 11 | MSI | 223 | 221 (99) | 176 (79) | 45 (20) |
| 20 | MSI | 30 | 29 (97) | 3 (10) | 26 (87) |
| 5 | MSI | 10 | 7(70) | 1 (10) | 6 (60) |

**Table S2. The expression of miR-200b and laminin-γ2 in the tumor budding cells of the multiplex stained cases.**

MMP: mismatch repair protein; MSI: microsatellite stable; MSS: microsatellite instable; TBCs: tumor budding cells.

^a^ Percentage of total TBC count
