# Supplementary figures and images for: MicroRNA-200b is downregulated in colon cancer budding cells
Source: PLoS One. 2017 May 26;12(5):e0178564. doi: 10.1371/journal.pone.0178564 (PMC5446202; doi:10.1371/journal.pone.0178564)

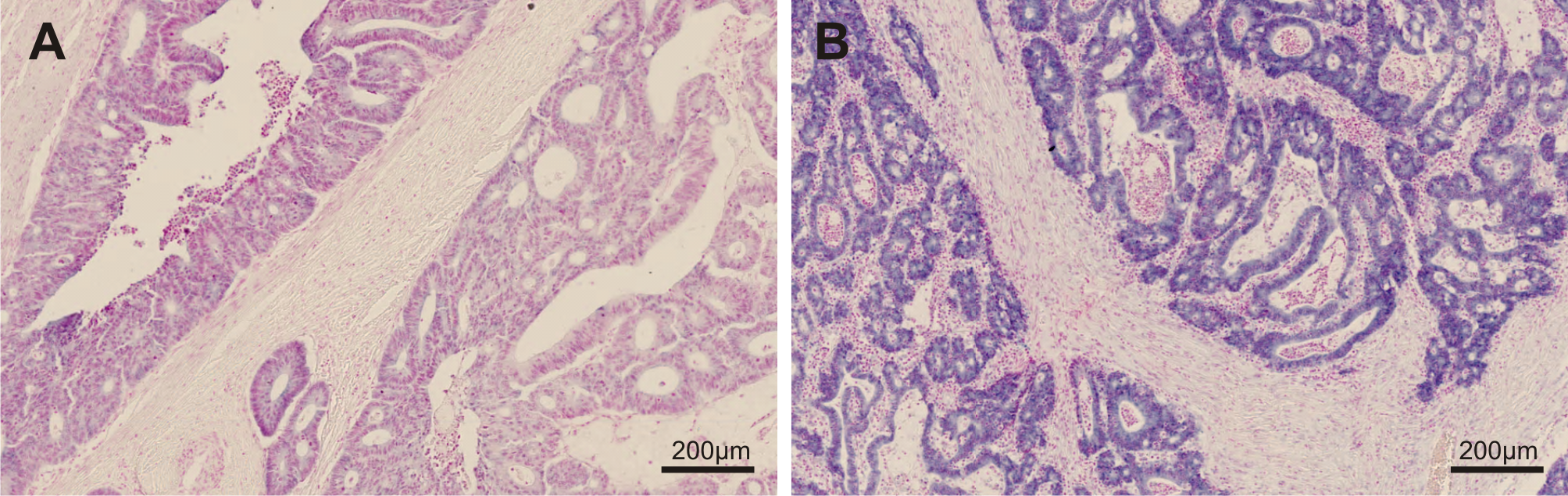

Supplement: S1 Fig — (A) Low miR-200b expression and (B) high miR-200b expression in the epithelial carcinoma cells of colon cancer. (TIF) [file pone.0178564.s003.tif]

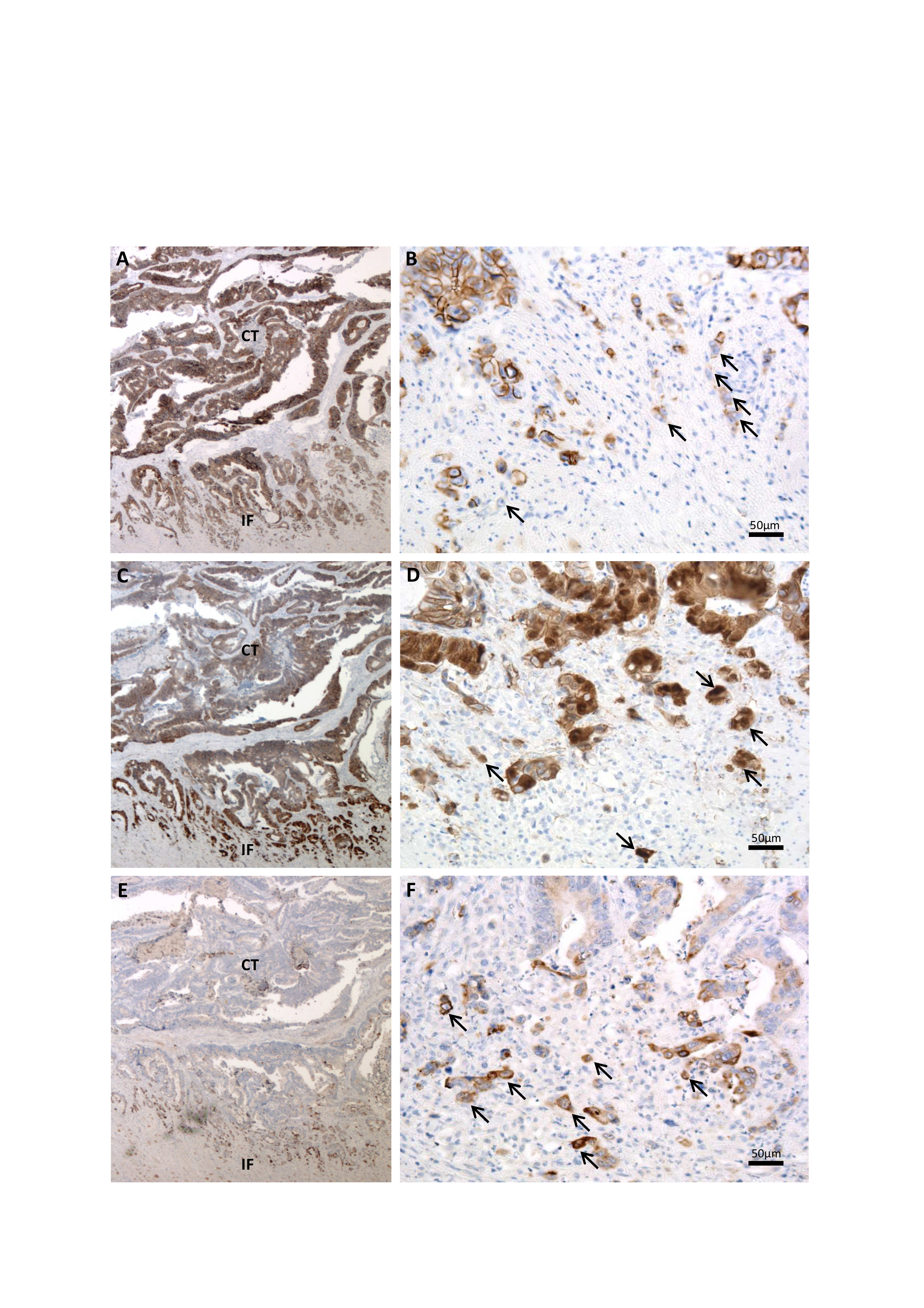

Supplement: S2 Fig — (A) Declining E-cadherin expression from central tumor (CT) towards the invasive front (IF) where (B) tumor buds show decreased membrane expression (arrows). (C) β-catenin expression changes from a chiefly membranous and cytoplasmic pattern to (D) nuclear localization in the tumor buds (arrows), while (E+F) laminin-5γ2 is upregulated at the invasive front and the tumor buds (arrows). (TIF) [file pone.0178564.s004.tif]
